# Fusion rates support wired allograft combined with instrumented craniocervical fixation in the paediatric population

**DOI:** 10.1007/s00701-020-04287-9

**Published:** 2020-03-24

**Authors:** Justus L. Groen, Wilco C. Peul, Willem Pondaag

**Affiliations:** 1 Department of Neurosurgery, Leiden University Medical Center, PO Box 9600, 2300RC Leiden, The Netherlands; 2 Department of Neurosurgery, Haaglanden Medical Center, The Hague, The Netherlands

**Keywords:** Allograft, Bone fusion, Children, Craniocervical instability, Surgical technique

## Abstract

**Background:**

Occipitocervical and atlantoaxial instability in the pediatric population is a rare and challenging condition to treat. Variable surgical techniques have been employed to achieve fusion. The study aimed to assess bony fusion with rigid craniocervical fixation using an allograft bone block to serve as scaffold for bony fusion.

**Methods:**

This is a single center case series from a tertiary referral neurosurgical center. The series includes 12 consecutive pediatric patients with rigid craniocervical fusion between 2006 and 2014. The primary outcome was bony fusion as assessed by computed tomography and flexion-extension radiographs. The authors did not receive external funding for this study.

**Results:**

Twelve patients (age 1–15 years) were operated with a median imaging follow-up time of 22 months (range 6–69 m). A modified Gallie fusion technique with a tightly wired allograft bone block was used in 10 of 13 procedures. One patient underwent re-fixation due to screw breakage. Eleven out of 13 procedures resulted in a stable construct with bony fusion. All 10 patients operated with the modified Gallie fusion technique with sublaminar wiring of allograft bone block had bony fusion. No post-operative complications of the posterior fixation procedure were noted.

**Conclusions:**

The modified Gallie fusion technique with allograft bone block without post-operative immobilization achieved excellent fusion. We conclude there is no need to use autograft or BMPs in craniocervical fusion in the pediatric population, which avoids related donor-site morbidity.

**Level of evidence:**

Level IV—case series; therapeutic.

## Introduction

Occipitocervical (OC) and atlantoaxial (AA) fusion in children may be indicated to treat craniocervical instability resulting from developmental, congenital, inflammatory, traumatic, and neoplastic disorders. Craniocervical instability in children is extremely rare, although in some subpopulations like children Down syndrome [18], the incidence of symptomatic atlantoaxial subluxation is relatively high at 1–10%. Congenital occipitocervical instability may result from a variety of bony or soft-tissue abnormalities, including condylar dysplasia, odontoid dysgenesis, and ligamentous laxity.

Without treatment, “deformity begets deformity” resulting in craniocervical kyphosis and progressive instability leading to nervous system damage by impingement of the high cervical spinal cord and brainstem. Tetraparesis, swallowing and breathing problems, and even sudden death might be the devastating outcome in this young population.

Over the last decades, variable surgical techniques have been employed to achieve fusion of the cranial-cervical junction in children. Generally, one can distinguish (1) non-rigid techniques like external fixation (halo-vest immobilization); (2) internal fixation using posterior wiring and onlay bone only; and (3) variable techniques of internal rigid fixation: occipital plate to C-1 lateral mass screws and C-2 pars or pedicle screws (Harm’s modified Goel technique), C-2 laminar screws, transarticular screw placement (Magerl technique), combinations [9] or unilateral occipital cervical fixation constructs. [14] These techniques all need supplemental bone onlay to promote fusion. Most authors employ autograft from patient’s rib, iliac crest, or local bone, with or without addition of recombinant human bone morphogenetic protein (BMP) or bone marrow aspirate (BMA). The use of autograft in the pediatric population, however, may be limited due to the small size of harvest site. Besides that, harvesting of autograft will result in donor site morbidity, especially pain. In the present study, we describe a single center case series of rigid cranial cervical fusion using allograft instead of autograft.

## Materials and methods

### Patient population

All AA and OC fusions performed in consecutive pediatric patients in a 10 year cohort were reviewed in a tertiary referral clinic for complex spine conditions. The most common condition was a C1-C2 instability related to Down syndrome. All children except the one patient with a C1 fracture exhibited clinical pyramidal signs of spinal cord compression. Clinical data and details of surgery were gathered; clinical and radiological outcomes were retrospectively reviewed. All patients were subject to CT scan and if indicated a dynamic fluoroscopy at follow up. As this concerns a retrospective chart study, approval of the Medical Ethics Committee is not mandatory.

### Operative technique

We did not employ neurophysiological monitoring. Internal rigid fixation was performed by the senior authors in all patients using a screw and rod system. When local anatomy allowed for placement of a screw in C1, this was the preferred treatment option. Either lateral mass screws were placed in C1, used in two patients (ID3 and ID12), or transarticular C1-C2 screws in three patients (ID2, ID3-r, ID10). If placement of a screw in C1 was considered unsafe due to small size of the C1 anatomy, a craniocervical construct was chosen, and the C1 arch was fastened to the rod with a titanium cable (8 patients). For C2 fixation, we used screw techniques depending on local anatomy: pars interarticularis screws (9/13), pedicle screws (1/13) (as defined by Benzel [5]), or transarticular screws (3/13), typically 3.5–4.5 mm in diameter and 12-18 mm in length. If indicated, a small-sized occiput plate was fixed on the planum nuchae using four bicortical screws close to midline and connected with titanium rods. In four patients (ID 6, 7, 9, and 10), instrumentation of C3 and C4 was performed.

**Fig. 1:**
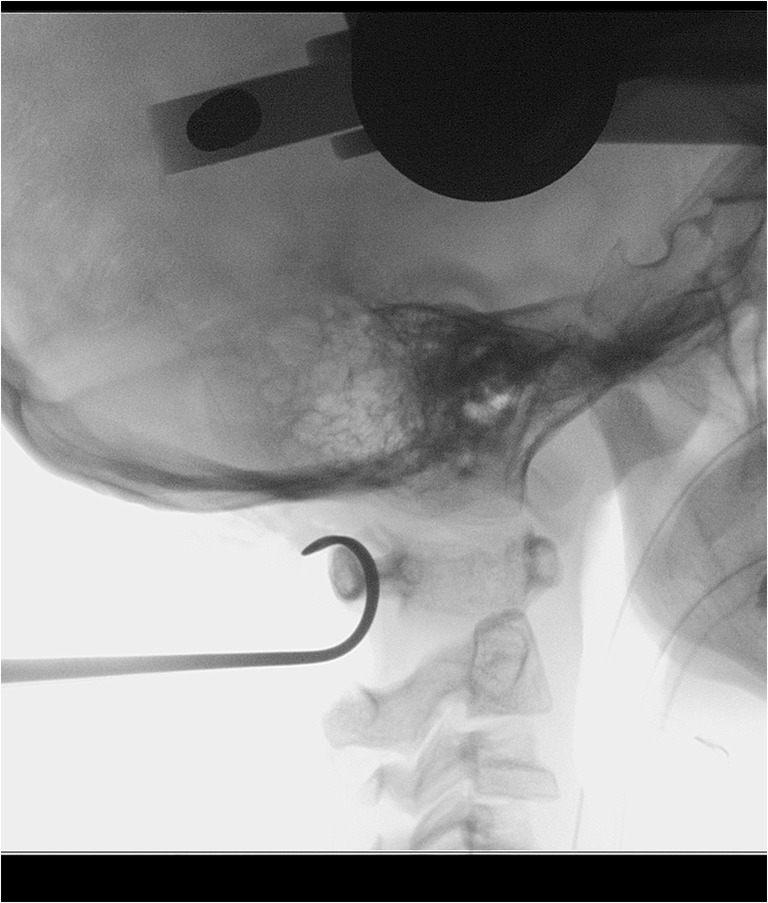
Fluoroscopy showing a Deschamps needle passed under the C1 arch to place a sublaminar wire

**Fig. 2:**
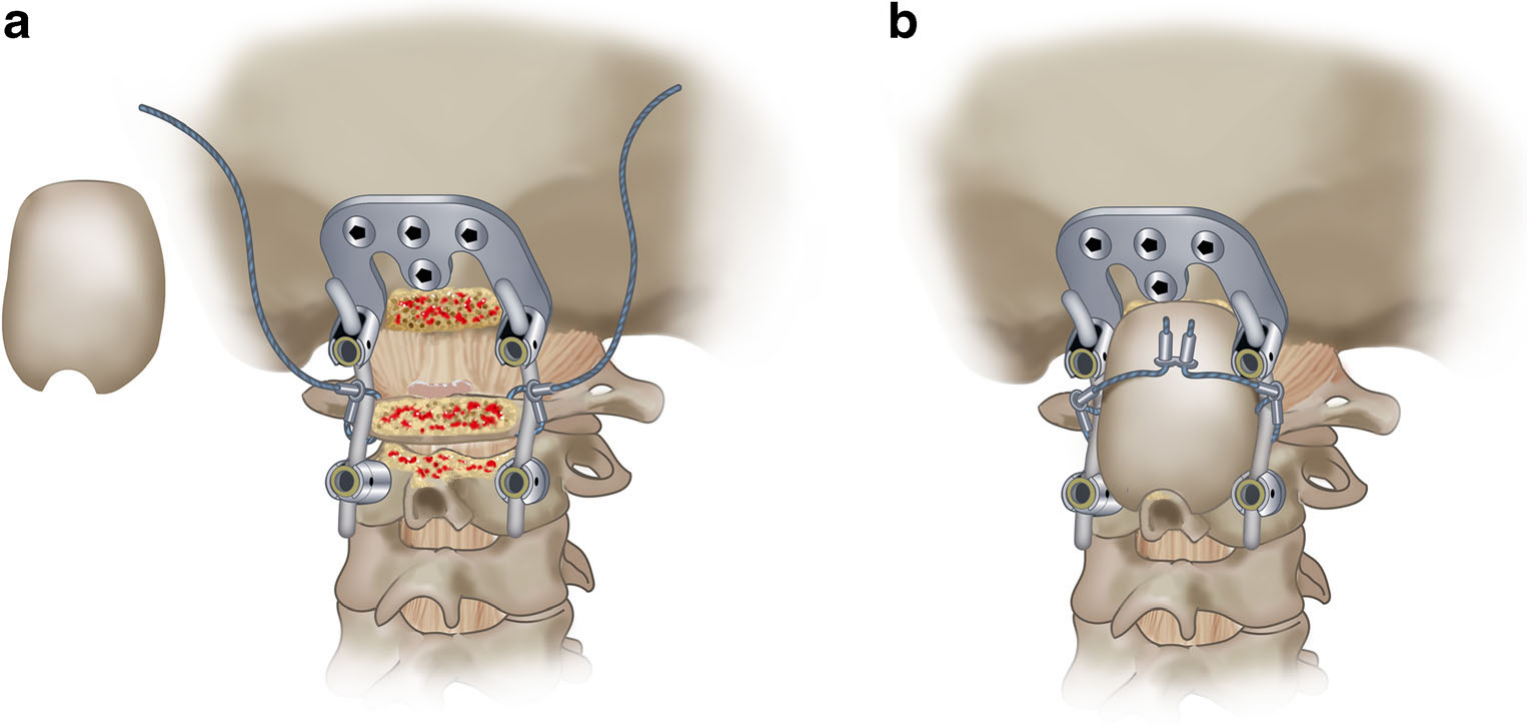
Drawing of the fusion technique. Frequently, the construct consisted of a C2 screw and an occiput plate. Before the rod-and-screw construct was put into place, a titanium cable was put around the C1 arch laterally on both sides. The titanium cable was wrapped around the rod, tightened, and crimped bilaterally with the system connector. The surface of the occiput, the C1 arch, and the C2 arch were partially decorticated with a high speed drill. The crafted bone graft was placed with good contact to the occiput, the C1 arch and, the C2 arch and spinous process, with the cortical surface posteriorly. The cable ends were then passed over the graft, tightened, and crimped using a double connector, and cut short

**Fig. 3:**
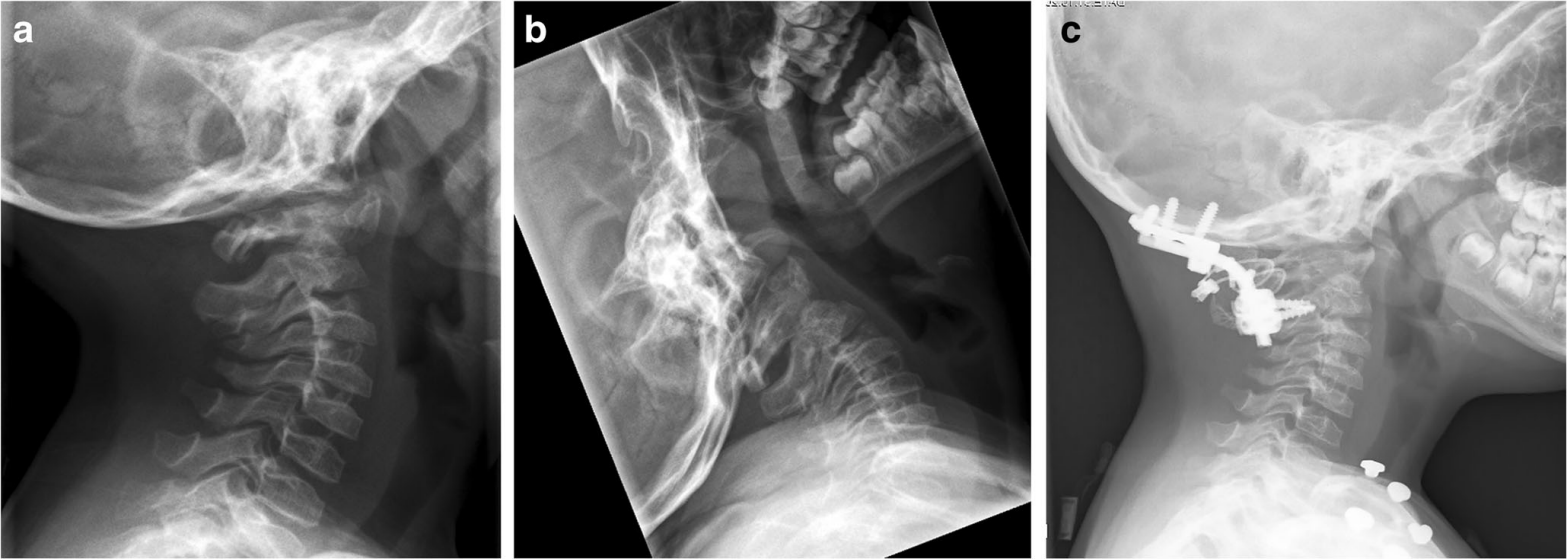
Fluoroscopy of patient ID-8. **a**/**b** Pre-operative ante-position of C1, mobile segment with posterior movement of C1 in extension. **c** Post-operative alignment of C1 after wiring of C1 arch and bilateral rods

We used a modified Gallie fusion technique. [23] Occipital bone surface, the posterior arch of C1, the C2 lamina, bilateral facet joints, and the spinous processes were decorticated with a high-speed drill before placing the allograft. Frozen allogeneic grafts were used as scaffold for bony fusion. Living donor femoral heads were employed, supplied by ETB-BISLIFE, a non-profit tissue institution (Galileiweg 8, Leiden, The Netherlands). A bone block was crafted to exactly fit the local anatomy using a bone rongeur and/or high speed drill. The monocortical bone block was carved from the femoral head, with a tight fit of the grafts spongious side to the posterior surface of the occiput and/or C1 posterior arch with a caudal notch to fit around the C2 spinous process. Using a Deschamps needle, a double titanium cable (Atlas Cable, Medtronic, Minneapolis, USA) was passed sublaminarly under the C1 arch (Fig. 1). Sublaminar wires under the C2 arch were not employed. In most patients, both cables were additionally guided around the rods of the screw and rod system bilaterally after which the cables were tensioned and crimped (Fig. 2). In this fashion, the C1 arch could be retracted posteriorly if indicated, and the resulting construct was considered stiffer including also C1 (Fig. 3). The cable was subsequently passed over the cortical side of the onlay bone block. Once the graft was tightly placed, the titanium cable was tensioned to 35 pounds and crimped on top of the bone block. Around the bone block graft, morcellized spongious graft was deposited, obtained from the remainder of the femoral donor head. In patient ID3, sublaminar wiring was not employed during the first surgical procedure, as the bone block seemed tightly fit between the occiput and C2 spinous process without wiring. In another two procedures (ID1 and ID2), only morcellized graft was inserted. Autologous bone marrow aspirate, aspirated from the patient’s iliac wing, was applied around the graft material in 4 patients. BMPs were not used (Table 1).

**Table 1:** Patient characteristics, surgery specifics, and fusion details of 12 pediatric patients with craniocervical instability

Patient			Diagnose		Surgery							FU imaging	
ID	Sex	AaS	Pathology	Etiology	Transoral	Strut	Wiring	BMA	Occiput fixation	C1 fixation	C2 fixation*	Months	Fusion
1	M	8	C1-C2 slip	Unknown	n	n	NA	n	Small plate, 4 BCS	None	Bilateral pars	6	Nonunion, stable construct
2	M	14	C1-C2 slip	Down syndrome	n	n	NA	n	2x small plate, 4 BCS	Unilateral TA	Unilateral TA	36	Union, C0–C2
3	M	13	C1-C2 slip, OO	Down syndrome	y	y	n	n	Small plate, 4 BCS	Bilateral lateral mass	Bilateral pars	12	Nonunion, screw #
3-revision	M	15	C2 screw #	Down syndrome	NA	y	y	y	NA	Sublaminar wiring	Unilateral TA	13	Union C0–C2
4	F	8	C1-C2 slip	SED	n	y	y	y	Small plate, 4 BCS	Sublaminar wiring	Bilateral pars	7	Union C0–C2
5	M	7	C1-C2 slip	ZMYND11 mt	n	y	y	n	Small plate, 4 BCS	Sublaminar wiring	Bilateral pedicle	32	Union C0–C2
6	M	15	C1-C2 slip	Down syndrome	y	y	y	n	Medium plate, 4 BCS	Sublaminar wiring	Bilateral pars	15	Union C1–C4 (ant + post)
7	F	10	C1-C2 slip	Chiari type II	y	y	y	n	Small plate, 4 BCS	None	Bilateral pars	42	Union C0–C3 (ant + post)
8	F	7	C1-C2 slip, OO	SED	n	y	y	y	Small plate, 4 BCS	Sublaminar wiring	Bilateral pars	9	union C0–C2
9	M	13	C1-C2 instability	Marfan syndrome	n	y	y	n	Small plate, 4 BCS	Sublaminar wiring	Bilateral pars	12	Union C0–C3 (ant + post)
10	M	9	C1-C2 instability	Larsen syndrome	n	y	y	n	None	Sublaminar wiring, bilateral TA	Bilateral TA	69	Union C0–C3 (ant + post)
11	M	1	Clival-odontoid resection	Chordoma	y	y	y	n	2 BCS, Mersilene	Sublaminar wiring	Bilateral pars	11	Union C0–C2
12	M	6	C1#, diastasis	Trauma	n	y	y	y	Small plate, 4 BCS	Bilateral lateral mass	Bilateral pars	12	Union C0–C2

*AaS* age at surgery, *BMA* bone marrow aspirate, *OO* os odontoideum, *SED* spondylo-epifysaire dysplasie, *BCS* bicortical screw, *TA* transarticular

#Breakage

^*^Pedicle/pars as defined by Benzel [5]

### Assessment of fusion

Patients underwent standard clinically examination at 8 and 26 weeks post-operatively. For confirmation of fusion, computed tomography was performed in all patients and flexion-extension radiographs of the cervical spine if indicated. Fusion was defined as cortical union of the allograft and central trabecular continuity as proof for a complete bony bridge [22] between the posterior arch of C2 and the occiput (OC fusion) or posterior arch of C1 (AA fusion). The fusion was assessed independently from radiologists by the senior authors. Once bony fusion was ascertained, this was defined as radiological endpoint, and no more CT scans were obtained.

## Results

### Baseline characteristics

We identified twelve pediatric patients with craniocervical instability who were treated with rigid posterior fixation and operated between 2006 and 2014. Mean age at surgery was 9.7 years (range 1–15 years), 25% of patients was female. All but one patient were over 5 years of age. All patients had instability of the C0-C1-C2 segment either as a direct result of their condition, or instability existed after surgery (e.g., tumor resection or transoral odontoid resection). Ten patients suffered from congenital malformations of the craniocervical junction: three in Down syndrome (with os odontoideum in one), two patients diagnosed with spondyloepiphyseal dysplasia congenital (SED), and the other patients were diagnosed with Larsen syndrome (*n* = 1), Marfan syndrome (*n* = 1), Arnold Chiari syndrome type 2 (*n* = 1), a syndrome caused by a de novo mutation in ZMYND11 [6] (*n* = 1), and a syndrome of unknown origin (*n* = 1). Two remaining cases suffered of acquired junctional instability: one due to traumatic fracture of C1 and one after transoral resection of a skull base chordoma. Transoral anterior decompression preceding the OC fixation was performed in 4 patients.

Median follow up imaging was 22 months (range 6–69 m). No post-operative complications of the posterior fixation procedure were noted. Median duration of admission was 6.6 days (range 4–11). Restriction of cervical motion by collar or halo frame in the post-operative period was not applied.

### Fusion rate

In the present cohort, 11/13 procedures resulted in a stable bony fusion; all primary surgeries that included a tightened wiring of allograft bone block resulted in bony fusion. Two of 13 occipitocervical fusion procedures resulted in a non-union (15%). Both non-unions occurred in patients without a wired bone block graft: patient ID3 showed a unilateral C2 screw breakage at follow-up imaging after 16 months as a result from persistent instability. A re-fixation was performed with allograft bone block, sublaminar wiring, and application of bone marrow aspirate, eventually resulting in a solid anterior and posterior C0–C2 fusion. In patient ID1, no bone block was placed. On follow-up imaging at 6 months, a stable position of the C1-C2 segment was seen, however, no bony union. Flexion-extension fluoroscopy shows no movement at follow-up time of 3 years; therefore, no re-intervention was indicated.

## Discussion

Bony fusion was achieved in all 10 children with craniocervical instability treated with a modified Gallie fusion technique using tightly wired allograft bone blocks. The two failures of fusion occurred in those two patients in whom such wired bone blocks were not employed. Based on this series, it is fair to conclude that there is no need to use autograft for craniocervical fusion in children, which avoids the post-operative donor-site pain and morbidity. Additionally, using rigid screw and rod fixation, post-operative immobilization is not needed, facilitating early rehabilitation. Although there has been some debate among spine surgery experts about the use of bone graft in craniocervical fixation, the current series with 2 failures by not using bone graft and our experience with pseudo-arthrosis in the adult population mandates the use of bone graft.

Limitations of the present study are the following: (1) our patient series is relatively small and includes diverse underlying pathology. This reflects the rareness of the indication for AA or OC fusion in the pediatric population. As our main outcome parameter was bony fusion, as the most appropriate outcome measure for the described surgical technique, we feel that the difference of the underlying pathology is not relevant. (2) In four patients, follow-up was less than 1 year, which is generally considered a minimum for reporting patient outcome. We consider, however, that once bony fusion has been demonstrated, that such can be regarded as irreversible end stage: the patient will not “unfuse.” Thus, for the current purpose to demonstrate the effectiveness of an allograft bone block to ensure bony fusion, the follow-up in our series can be considered adequate.

The use of allograft bone has several advances over autograft, especially in the pediatric population. Morbidity from autograft harvest site are well documented and frequent (9%), including post-operative pain, increased blood loss, increased infection risk, seroma formation, pelvic fracture, the risk of peripheral nerve injury, and donor site pain. Moreover, it is a challenge to harvest and craft a well-fitting bone block from a small costa or thin iliac crest in children. [8, 19, 21] Allografts are only osteoconductive, weakly osteoinductive, but not osteogenic like autografts; therefore, their use in posterior fixation is associated with a higher rate of nonunion. [4] An older publication by Koop et al. (1984) noted that pseudoarthrosis occurred in one patient who received allograft instead of autograft in their pediatric OC fusion procedures, strengthening this author’s view that autograft is superior in fusion. [12] In a recent meta-analysis on OC fusion in children, a strong surgeons’ preference for the use of autologous bone was shown, as it had been used in 539 pediatric cases compared with only 65 children where allograft had been employed using various fixation techniques. Higher fusion rates were seen with autologous bone graft compared with allograft (97% vs 85%). However, in a subgroup analysis for rigid internal fixation techniques including only 18 patients from 5 different studies where allograft was used, the differences were smaller (99% vs 94%, respectively). [19] In an adult population, Godzik and colleagues reported in adult population bony OC fusion in allograft group in 18 of 19 (95%) and 8 of 8 (100%) in the autograft group after a minimum of 12-month follow-up. [8]

To overcome the lack of osteoinductive function of allograft, the use of bone marrow aspirate (BMA) [15] or bone morphogenetic proteins (BMP) are advocated. Some case series in lumbar fusion in adults suggest that addition of BMA on an allograft scaffold might improve bony fusion [11, 16, 24], however, as yet no studies in cervical posterior fixation in children are available. In our series, we used BMA on the site of fusion in 4/13 procedures. BMP is routinely employed in most reports of OC and posterior cervical fusion in adults with allograft. However, in a recent meta-analysis, Reintjes et al. did not find a statistically significant association of BMP with successful fusion in any of the univariate or multivariate analyses. [19] Sayama also concluded there is no need for routine use of recombinant BMP in the pediatric age group. [20] Based on our current findings and the literature, we do not consider application of BMP to be essential for fusion if rigid fixation is achieved.

Essential for fusion is an adequate fit of the onlay bone block and proper surgical preparation of the posterior surfaces for optimal bone-to-bone contact. A second requisite for fusion is mechanical stress on the bone-to-bone surface, aka as Wolff’s law. [2] With rigid fixation, there is a risk that the bone is shielded from mechanical stress by the stiffness of the screw and rod system, which may result in resorption of the graft. [17] In this series, we described the use of allografts tailored to fit between the occiput or the C1 arch and C2 spinous process and compressed with tensioned wiring around the lateral rods, the C1 arch and/or the C2 spinous process. We believe that tensioning of the graft is essential to achieve bony fusion. On this account, we show with the present series that fusion rates are excellent by using the described technique. For sublaminar wiring, the Deschamps needle [7] proved to be a very useful instrument.

Some authors reserve rigid instrumentation for children above 5 years of age, or even above 10 years, based on report on abnormal cervical spine growth in children who underwent craniocervical stabilization at young age. [1, 3, 10] In our series, six patients underwent surgery under the age of 10 years, and all showed bony fusion and no growth difficulties (mean follow-up interval 22.5 months), suggesting that internal rigid fixation is feasible in this population too. In these very young children, the surgical challenge can be considered substantially more profound than in children above 10 years. The youngest patient was a 1-year-old boy (ID11) in whom we performed a C0–C2 fixation after transoral clival chordoma resection. At follow-up imaging at 11 months, there was bony fusion in adequate position. Follow-up may be too short to appreciate growth abnormalities in some of the patients in present series; however, Martinez-del-Campo and colleagues described normal growth, curvature, and alignment parameters in their series of children with OCF constructs. [13] The retrospective nature, the small number of patients and single center character of this cohort study make it uncertain that our results can be extrapolated to all pediatric patients who need a craniocervical fusion. We feel, however, to have made a strong argument in favor of the described technique.

## Conclusion

This report on surgical strategy differs from other reports on craniocervical fusion in the pediatric population. With the presented modified Gallie fusion technique using allograft (instead of autograft) bone block and compressed wiring technique (both wiring to the fixation rods and sublaminar wiring), we achieved a rigid construct and excellent AA and OC fusion rates. Both the use of BMPs and post-operative external immobilization are not indicated in these children.
